# Impact of a Plant Sterol Food Supplement on Eryptotic and Associated Cardiometabolic Parameters: A Randomized Placebo-Controlled Trial in Statin-Treated Patients

**DOI:** 10.3390/foods13244108

**Published:** 2024-12-19

**Authors:** Diego Miedes, Raquel Ortega-Luna, Sonia Broseta, Sergio Martínez-Hervás, Ángeles Álvarez-Ribelles, Víctor Collado-Díaz, Antonio Cilla, Amparo Alegría

**Affiliations:** 1 Nutrition and Food Science Area, Faculty of Pharmacy and Food Sciences, University of Valencia, 46100 Valencia, Spain; diego.miedes@uv.es (D.M.); sonia.broseta@uv.es (S.B.); amparo.alegria@uv.es (A.A.); 2 Department of Pharmacology, Faculty of Medicine, University of Valencia, 46010 Valencia, Spain; raquel.ortega-luna@uv.es (R.O.-L.); angeles.alvarez@uv.es (Á.Á.-R.); victor.collado@uv.es (V.C.-D.); 3 Endocrinology and Nutrition Department, Hospital Clínico Universitario, 46010 Valencia, Spain; sergio.martinez@uv.es; 4 Department of Medicine, University of Valencia, 46010 Valencia, Spain; 5 INCLIVA Institute of Health Research, 46010 Valencia, Spain; 6 CIBER Diabetes and Associated Metabolic Diseases (CIBERDEM), 28029 Madrid, Spain; 7 CIBER Enfermedades Hepáticas y Digestivas (CIBERehd), 28029 Madrid, Spain

**Keywords:** hypercholesterolemia, erythrocytes, endothelium adhesion, atherosclerosis, phytosterols

## Abstract

Eryptotic erythrocytes are prone to adhere to the vascular endothelium, provoking atherosclerosis. As statins do not prevent eryptosis compounds with anti-eryptotic effects could help treated hypercholesterolemic subjects in decreasing cardiovascular disease risk. Plant sterols (PSs) have shown this anti-eryptotic effect ex vivo, along with their cholesterol-lowering activity. A parallel double-blind placebo-controlled randomized trial was conducted using a PS-food supplement (2 g of PS/day) (case, n = 13) or a placebo supplement (control, n = 13) in statin-treated hypercholesterolemic subjects. Blood samples were extracted before (T0) and after (T1) a 6-week treatment, and erythrocytes were isolated for biochemical determination, phosphatidylserine externalization (EPHS), cell size and reduced glutathione (GSH) analyses, and endothelium adhesion evaluation. A reduction in glucose (4.3%) and LDL cholesterol (9.2%) was observed only in the control group, whereas in the case group, an increase in ApoA1 (6.4%) was observed. Neither EPHS, cell size nor GSH were modified by the treatment with any of the supplements, whilst endothelium adhesion was reduced (55.1%) only in the case group. These results suggest that the PS supplement may improve some cardiovascular health parameters in the target population even though eryptosis status is not modified by this treatment.

## 1. Introduction

The suicidal death of erythrocytes, also known as eryptosis, is a normal recycling process of the blood. Oxidative stress, osmotic shock, and energy depletion are responsible for targeting eryptosis. Likewise, some drugs and xenobiotics can effectively activate the process as well. The eryptotic event is characterized by intracellular calcium increase, cell shrinkage, ceramide accumulation, and, mainly, the externalization of phosphatidylserine (EPHS) to the outer layer of the cell membrane [1]. The disrupted membrane of red blood cells (RBCs) is prone to adhere to the vascular endothelium and platelets through the CXCL16/SR-PSOX receptor [2,3]. In this sense, an increased eryptosis leads to higher adhesion to the endothelium, an increase in the inflammatory status, and thus, atherosclerosis, which is strongly related to worse cardiovascular (CV) health [4]. Additionally, eryptosis has been associated with metabolic syndrome, another risk factor of CV disease [5].

Hypercholesterolemia has been associated with lower antioxidant status, higher eryptosis levels, and higher adhesion to the endothelium. This is biologically plausible since high levels of plasmatic cholesterol are directly responsible for atherosclerosis. However, the traditional pharmacologic treatment with statins does not seem to protect against eryptosis and evoked even superior EPHS than non-treated subjects [6]. In fact, dyslipidemic atorvastatin users have also previously shown higher eryptosis levels than healthy subjects [7], a finding that had been observed ex vivo with atorvastatin [8], but also with simvastatin [9]. This could explain why some pharmacologic-treated patients with in-range low-density lipoprotein cholesterol (LDL-c) levels still suffer CV events more than the healthy population.

For this reason, the search for compounds capable of reducing or maintaining the EPHS is of paramount relevance. In this sense, plant sterols (PSs) present potential as anti-eryptotic compounds. Nevertheless, the effect of PS on eryptosis has only been evaluated ex vivo, with erythrocytes from eight volunteers. These erythrocytes were stress-induced with tBOOH 75 μM, and the protection of a mixture of PS at physiological serum concentration was evaluated (22 μM, compatible with a normal intake of PS-enriched foods). PSs were able to protect the RBC against oxidative stress by increasing the levels of reduced glutathione (GSH). The intracellular calcium was also reduced. These results suggest that PS may protect RBC from an increased EPHS [10]. Additionally, PS are compounds with widely recognized cholesterol-lowering activity. PS can exert up to a 12.5% reduction in the total plasma cholesterol (TC) with intakes from 1.5 to 3 g per day with enriched yellow fat spreads, dairy products, mayonnaise, and salad dressings [11]. In this line, previous studies from our research group have shown that the intake of PS (2 g/day with enriched milk-based fruit beverage) can reduce TC and exert an anti-inflammatory effect on hypercholesterolemic post-menopausal women [12,13]. Inflammation and oxidative stress are key processes in the development of atherosclerosis, increasing oxidized LDL-c, which stimulates the pro-inflammatory and pro-thrombotic activity of macrophages [14]. In addition, several randomized controlled trials (RCTs) have evaluated the effect of PS intake (1.8 to 6 g/day from 4 to 85 weeks) on lipid markers in hypercholesterolemic statin-treated patients to assess the potential synergic effect, 2 and 2.5 g/day being the most common doses. The additive effect of PS on TC and LDL-c lowering (0.3 mmol/L in both markers) without affecting high-density lipoprotein cholesterol (HDL-c) levels represents a positive synergic effect [15]. It is worth noting that only 1 out of 15 RCTs was conducted using a PS supplement [16]. Therefore, taken together, these findings it is hypothesized that PS may serve as a preventive dietary approach for CV disease-risk factors (moderate hypercholesterolemia and eryptosis) with beneficial or synergistic effects when used alongside statin therapy.

To date, no studies have evaluated the effect of PS supplementation on the eryptotic parameters of statin-treated hypercholesterolemic patients. For this reason, this study aims to assess the effect of the chronic intake of a PS-enriched food supplement on the eryptotic status and the associated cardiometabolic and adhesion to the endothelium parameters in hypercholesterolemic statin-treated subjects.

## 2. Materials and Methods

### 2.1. Plant Sterol-Enriched Food Supplement

A powdered food supplement enriched with 2 g of PS and an analogous placebo supplement without PS were manufactured (NutraResearch 2011 SL, Hospitalet de Llobregat, Spain). For this purpose, free microencapsulated PS powder from tall oil (Lipophytol^®^ P Dispersible, Lipofoods, Barcelona, Spain) was added to the PS-enriched food supplement. As excipients, mannitol, citric acid, magnesium stearate, hydrated silica, sucralose, and lemon flavoring were used for both food supplements (enriched and placebo). The manufacturing conditions were similar for them, having the same appearance, but with different anonymous labeling.

In a previous test for flavoring selection, 4 volunteers were chosen. They tested 3 different flavors (lemon, orange, and berries) of both enriched and non-enriched supplements, and were told to judge the smell, taste, and solubility. In the end, the lemon flavor was selected as the most pleasant.

### 2.2. Study Design

A parallel double-blind, placebo-controlled clinical trial was designed. Twenty-six subjects were contacted, interviewed, and selected (between September 2023 to May 2024) to participate in the study, and if they complied with the inclusion criteria, were sequentially numbered. All had to be subjects with mild hypercholesterolemia diagnosed according to the guidelines of the European Society of Cardiology/European Atherosclerosis Society [17] (LDL-c ≥ 160 mg/dL at the time of diagnosis) under treatment with statins of low-to-moderate intensity (atorvastatin: 10–20 mg; simvastatin: 20–40 mg; rosuvastatin: 5–10 mg; pitavastatin: 1–4 mg) prior to the selection process and an age range of 18–70 years. The exclusion criteria were smokers; previous episodes of cardiovascular disease; diabetes mellitus; subjects in secondary prevention; liver or renal disease; uncontrolled hypothyroidism; consumption of PS-enriched foods or supplements; other analytical abnormalities or previous illnesses; or treatment with lipid-lowering drugs other than the aforementioned ones. The participants (n = 26) were randomly divided into 2 groups (Random Allocation Software 2.0). Case subjects took a daily food supplement enriched with 2 g of PS (n = 13, 7 females, 64.7 ± 6.0 years, and 26.9 ± 2.3 kg/m^2^); while control subjects took a daily food supplement without enrichment (n = 13, 8 females, 59.7 ± 8.4 years, and 27.2 ± 3.4 kg/m^2^). Both groups of subjects were told to take it before the main meal for 6 weeks.

The adherence to the treatment (number of non-ingested sticks and changes in the suggested schedule of intake), sensorial (ease of taking and acceptance), and adverse effects (bloating, fullness, indigestion, altered bowel habit, and/or any other indicated by the participants) were evaluated with a questionnaire conducted at the end of the study.

The study design and protocol were made according to the Declaration of Helsinki and were approved by the Ethics Committee of the Hospital Clínico Universitario de Valencia (approval number 2023/069) and registered in the Clinical Trials database (ClinicalTrials.gov ID: NCT05901246). All the participants received and signed an informed consent.

### 2.3. Blood Samples Collection

Each participant was subjected to 2 blood extractions, one before starting the treatment (T0) and one after the 6-week intervention (T1). The venipuncture was performed after overnight (10–12 h) fasting in the Endocrinology and Nutrition department of the Hospital Clínico Universitario de Valencia. A single tube was taken for the biochemical determination and 3 K_2_EDTA tubes were taken for both the eryptosis and adhesion assays. For this purpose, immediately after blood extraction, erythrocytes were isolated from the plasma and buffy coat by centrifugation (180 g/10 min/4 °C) and washed with PBS, repeating this process 3 times to fully isolate the red blood cells (RBCs). A solution of 0.4% *v*/*v* hematocrit in Ringer solution (125 mM NaCl, 5 mM KCl, 1 mM MgSO_4_, 32 mM HEPES, 5 mM glucose, and 1 mM CaCl_2_) (0.4% RBC) was prepared for the subsequent assays, as described by Cilla et al. [6].

### 2.4. Determination of Biochemical Parameters

One blood sample aliquot was used for the determination of the biochemical cardiometabolic parameters. Glucose, TC, triglycerides, HDL-c, Apo A1, Apo B, hs CRP, and insulin were measured using standardized and validated methods as previously described [18]. The LDL-c was calculated by Friedewald’s formula. The HOMA index was calculated using the following formula: (Glucose × 0.0555 × insulin)/22.5.

### 2.5. Evaluation of Phosphatidylserine Externalization (EPHS) and Forward Scatter (FSC)

In order to determine the degree of evolution of the eryptotic event, the EPHS to the outer layer of RBCs was measured [19]. For this purpose, 35 μL of 0.4% RBC were mixed with 100 μL of PBS, centrifuged (145 *g*/5 min/25 °C) to wash the sample, and once the supernatant was discarded, the pellet was suspended in 100 μL binding buffer prior to annexin V-FITC addition (5 μL). The samples were incubated in darkness for 15 min. Then, 400 μL of binding buffer was added and fluorescence was measured by flow cytometry (ʎ_exc_ = 488 nm and ʎ_em_ = 527/532 nm; FACS Verse, BD Biosciences). The cell volume was measured using the geometric mean of the forward scatter (FSC). At least 1 × 10^4^ events were analyzed for each sample.

### 2.6. Intracellular Glutathione

The content of GSH in the erythrocytes was determined by flow cytometry using 5-chloromethylfluorescein diacetate (CMFDA), a specific dye that binds nonprotein cytosolic thiol [6]. Briefly, 55 μL of 0.4%RBC were mixed with 445 μL of PBS and incubated (40 min, 37 °C, 5% CO_2_, and 95% relative humidity). After centrifugation (145 g/5 min/25 °C), the cells were suspended in 500 μL of PBS and fluorescence was measured by flow cytometry (ʎ_exc_ = 488 nm and ʎ_em_ = 527/532 nm). At least 1 × 10^4^ events were analyzed for each sample.

### 2.7. Assessment of the Adhesion to the Endothelium

Human umbilical vein endothelial cells (HUVECs) were freshly isolated from umbilical cords provided by healthy female donors from the Hospital Clínico Universitario de Valencia, under informed consent approved by the hospital’s ethics committee (approval number 2021/038), through collagenase type I treatment. Briefly, the umbilical cord veins were rinsed of blood products with warm PBS, after which the vein was filled with collagenase (1 mg/mL) for 17 min at 37 °C. The cords were then gently massaged to ensure the detachment of endothelial cells from the vessel wall. The digest was collected, centrifuged, and pelleted once more. The pellet was re-suspended in endothelial cell growth medium (EGM-2) in T25 culture flasks, where the cells were cultured until confluence.

The erythrocyte–endothelium cell adhesion assay was performed by using the parallel-plate flow chamber technique [6,20]. Passage 1 from these primary cultures was subsequently employed. After reaching confluence, the primary cultures were detached with trypsin and transferred to fibronectin (5 μg/mL)-coated 25 mm plastic coverslips on a 6-well plate until confluence (approximately 48 h). RBCs (5 × 10^6^ cells/mL) were re-suspended in Dulbecco’s PBS containing 20 × 10^−3^ M HEPES and 0.1% human serum albumin. To perform the adhesion assays, the coverslip containing confluent HUVEC monolayer was placed in the flow chamber. RBC suspensions were drawn across the monolayer at a flow rate of 0.37 mL/min (approximately shear stress of 0.7 dyne/cm^2^) for a period of 3 min, after which the flow was stopped for 1 min and re-started for another 2 min (total time recorded: 6 min). The flow chamber was on a microscope (Nikon Eclipse TE 2000-S, Nikon, Amstleveen, the Netherlands) connected to a video camera (Sony Exware HAD, Koeln, Germany) where images were recorded. Adhesion was measured by counting the number of cells that maintained stable contact with the HUVEC monolayer for 30 s.

### 2.8. Lifestyle Surveys

A qualitative differentiation regarding diet and exercise was performed by using validated surveys, both only at T0 of the trial using the Mediterranean Diet Adherence Screener (MEDAS) questionnaire [21] and Physical Activity Questionnaire-short form (IPAQ-short) [22].

### 2.9. Statistical Analysis

Data are presented as mean ± standard deviation for each group. Shapiro–Wilk, D’Agostino, and Pearson normality tests were performed, and the possible presence of outliers was verified. Unpaired Student’s *t* test or Wilcoxon signed-rank test were used to determine statistically significant differences between the treatments (*p* < 0.05). Correlations between different variables were evaluated using the Pearson correlation coefficient, the Chi-square test, and Fisher’s exact test. GraphPad Prism 8.0.2 (GraphPad Software Inc., San Diego, CA, USA) was used for all the analysis.

## 3. Results

### 3.1. Biochemical Parameters

The results of the biochemical analysis before and after the treatment for both groups are shown in Table 1. In the control group, a statistically significant decrease (*p* < 0.05) was observed in the average contents of glucose (4.6%) and LDL-c (9.2%) after the intervention. In the case group, a significant increase (*p* < 0.05) in Apo A1 (6.4%) was observed.

### 3.2. Eryptosis and Redox Status

**Figure 1:**
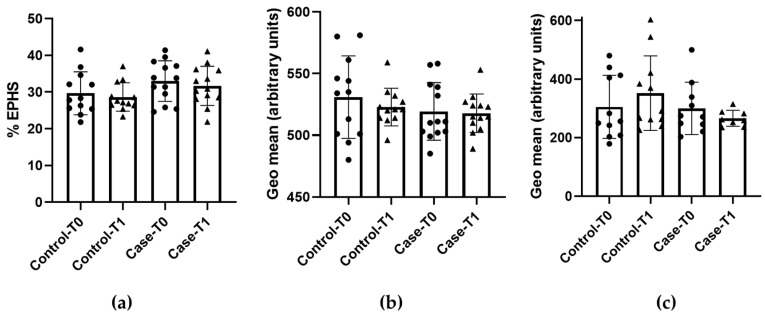
Eryptosis and redox status before (T0) and after (T1) the treatment with placebo (control) or plant sterol-supplement (case) (**a**) Percentage of cells expressing phosphatidylserine externalization (EPHS); (**b**) Relative size of cells (FSC); (**c**) Levels of reduced glutathione (GSH). Results are expressed as mean ± standard deviation (n = 3).

Data on the EPHS, FSC and intracellular GSH levels are shown in Figure 1. Regarding EPHS, the results show that no differences between the groups were observed. Neither the placebo (control) nor PS supplement (case) groups varied statistically (*p* > 0.05) after the 6-week treatment (control: T0, 29.7 ± 5.9 and T1, 28.7 ± 3.9% EPHS; case: T0, 32.9 ± 5.6 and T1, 31.7 ± 5.3% EPHS) (Figure 1a). The same output was observed for FSC since both treatments produced no differences in cell size after the 6-week period (*p* > 0.05) (control: T0, 531 ± 33 and T1, 523 ± 15; case: T0, 519 ± 23 and T1, 518 ± 16 geometric mean) (Figure 1b).

Regarding the redox status of the erythrocytes, after withdrawing the outliers detected in the GSH measurement, no difference was observed in both groups after the treatment period (*p* > 0.05) (control: T0, 305 ± 108 and T1, 352 ± 127; case: T0, 300 ± 89 and T1, 266 ± 27 geometric mean) (Figure 1c).

Considering these results, we decided to stratify them into homogenous groups based on sex, age (cut-off point > 60 years), body mass index (cut-off point > 27 kg/m^2^), and adherence to the treatment (maximum five unused portions). None of them showed statistically significant differences between groups (*p* > 0.05), confirming that the distribution of data were homogenous).

### 3.3. Adhesion to the Endothelium

**Figure 2:**
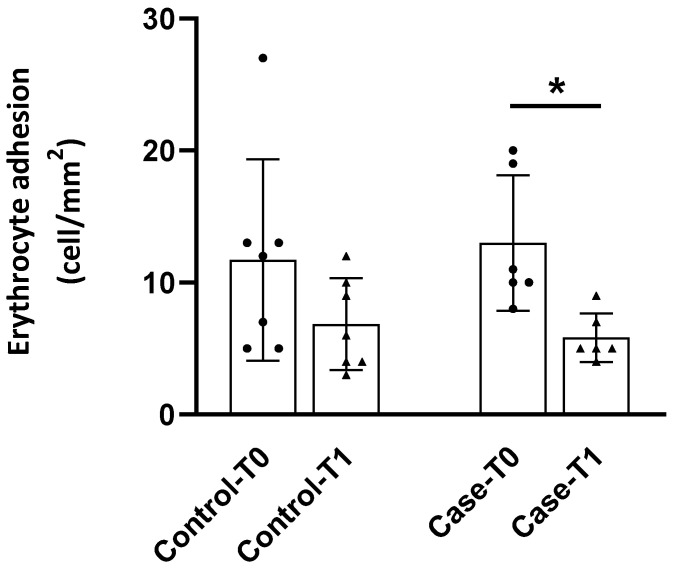
Adhesion of erythrocytes of hypercholesterolemic patients to the vascular endothelium before (T0) and after (T1) the treatment with placebo (control) or plant sterol-supplement (case). The results are expressed as mean ± standard deviation. * indicates differences between the basal and final measurement *(p* < 0.05).

Subjects assigned either to the control or to the case group presented an adhesion of their erythrocytes to the vascular endothelium of 11.7 ± 7.6 cells/mm^2^ and 13.0 ± 5.1 cells/mm^2^, respectively, at T0 (Figure 2). After 6-week treatment, there was a significant (*p* < 0.05) reduction in this parameter in those patients that received the PS food supplement, but not in the ones to whom placebo was administered (Figure 2).

### 3.4. Lifestyle Surveys

Table 2 shows the adherence to the Mediterranean Diet and the physical activity level of the subjects from the study. None of them were classified as having low adherence to the Mediterranean Diet, showing that the population studied has generally good dietetic habits. Most of the subjects were classified as low physical activity (53.8%), while only three of them (11.5%) were considered as high physical activity. No differences were observed dividing by sex in the proportion of each level (*p* > 0.05).

### 3.5. Correlations Between Variables

**Figure 3:**
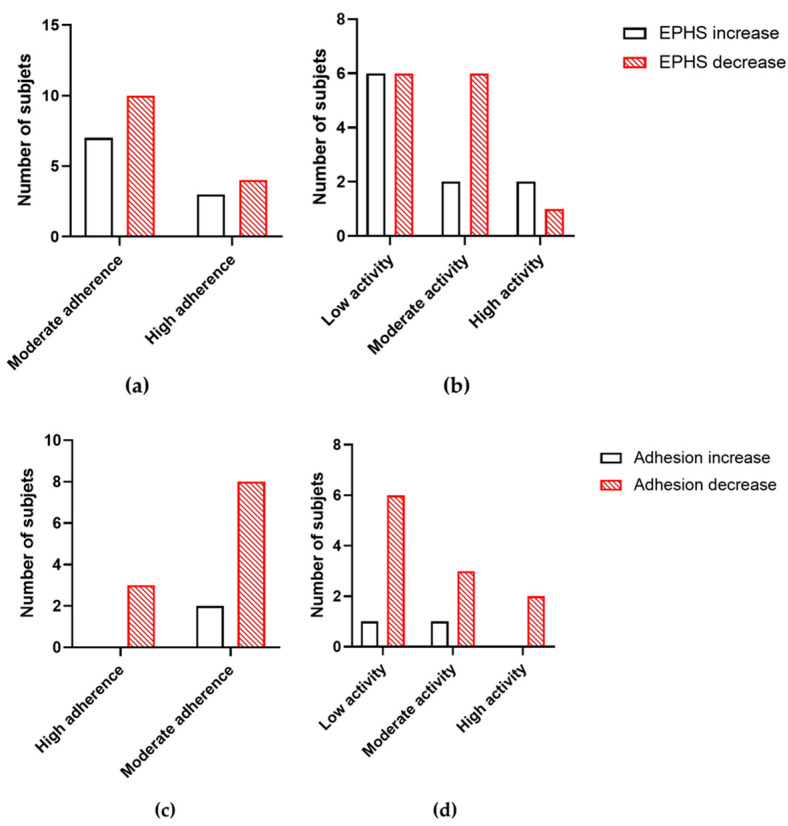
(**a**) Correlation between adherence to the Mediterranean Diet and the change in EPHS; (**b**) correlation between the level of physical activity and the change in EPHS. (**c**) Correlation between adherence to the Mediterranean Diet and the change in adhesion to the endothelium; (**d**) correlation between the level of physical activity and the change in adhesion to the endothelium. EPHS: externalization of phosphatidylserine.

The possible correlation between a qualitative variable and the main quantitative outputs (EPHS and RBC adhesion to the endothelium) was analyzed. Neither the adherence to the Mediterranean Diet nor the level of physical activity were able to predict the output of both parameters, showing no correlation (*p* > 0.05) (Figure 3). Nevertheless, the adhesion to the vascular endothelium and Apo A1 levels were considered for searching for any correlation, the variables being potentially linked with CV health. No correlation was observed between both variables at T0 (r = 0.156, *p* = 0.739 in the control subjects, and r = 0.230, *p* = 0.660 in the case subjects). At T1, however, the trend observed was the opposite in the control (r = 0.482, *p* = 0.518) vs. case subjects (r = −0.799, *p* = 0.410), showing a slight inverse correlation between adhesion to the endothelium levels and Apo A1 ones only after the treatment with the PS supplement.

## 4. Discussion

No reduction in TC or LDL-c was observed in the PS-food supplement group. The subjects included in this group were under statin treatment and consequently had relatively low baseline TC (172.2 mg/dL) and LDL-c (98.6 mg/dL) concentrations. Several studies have been carried out in hypercholesterolemic patients receiving statin therapy (atorvastatin, simvastatin, rosuvastatin, lovastatin, pravastatin, orpravastatin, or cerivastatin, in variable doses) and PS. The subjects received mostly 2 to 3 g per day of PS for at least 4 weeks to simulate a chronic intake. The only one combining statin treatment (40 mg of atorvastatin), and a PS food supplement demonstrated that the PS addition (2 g/day) was able to reduce 7.7% of TC and 6.5% of LDL-c compared to the statin alone [16]. This is a relatively low change when compared to the reduction stated in the regulations of the European Union for PS-enriched foods (up to 12.5% with 2.5–3 g/day of PS) [10]. Subjects that respond better to the statin treatment might benefit less from the activity of PS, since both have completely different mechanisms of action (statins only reduce the synthesis whereas PS only reduces the absorption). However, it must be pointed out that the study conducted by Malina and colleagues recruited 86 participants, enough to observe those slight differences [16] in comparison with our study which presented a relatively small number of participants as a main limitation.

Furthermore, previous RCTs from our group using similar PS ingredients also saw slight decreases in TC and LDL-c. In that case, the treatment was a PS-enriched milk-based beverage (2 g/day), and the duration of the treatment (6 weeks) was the same. The participants were not under pharmacologic treatment, resulting in notably higher baseline TC and LDL-c levels. Even so, the reduction in TC was only of 2.9 and 4.7%, and the reduction in LDL-c of 5.1 and 9.0% [12,13]. Basal levels affect the potential reduction produced by the PS. In this sense, higher baseline levels are significantly related to a higher decrease in TC and LDL-c [23,24]. Compared with these previous studies, the baseline levels of TC and LDL-c were ~ 25% and ~ 30% lower in our study.

Notwithstanding the previous observations, after treatment with PS-enriched supplement (case group), a significant increase (*p* < 0.05) in apolipoprotein ApoA-1 occurs. This protein is the main component of HDL lipoproteins, a known CV disease protective indicator. The HDL-c concentration itself may not correctly indicate the cholesterol efflux (the main function of these particles), but ApoA-1 does [25]. Indeed, ApoA-1 itself removes cholesterol from the atheroma plaque through lecithin-cholesterol acyltransferase (LCAT) activation. In addition, this protein can improve the atherosclerotic environment even more due to its anti-inflammatory effect. In keeping with this, ApoA-1 may increase nitric oxide synthesis, producing vasorelaxation [26]. Furthermore, this protein is capable of inhibiting the expression of CD11b, a protein from leukocytes that triggers inflammatory response, and is directly related to the adhesion of these cells to the vascular endothelium [27]. This could be interesting as an important finding in our study is the fact that a reduction in the adhesion to the endothelium of RBC in the case group was observed. Thus, it is easy to hypothesize that the increase in Apo-1 could be inhibiting not only the adhesion molecules of leukocytes—such as CD11b—but also of those of RBC or endothelium. The inverse correlation observed (r = −0.799) after the treatment with the PS supplement between ApoA-1 levels and RBC adhered to the endothelium suggests this mechanism of action. Although PSs do not directly affect the eryptotic status of the RBC (no changes in EPHS), they could exert an effect on the CXCL16/SR-PSOX or other endothelium receptors, explaining the reduced adhesion to the endothelium.

EPHS may increase with the intake of cholesterol-lowering drugs like statins, as stated before in this work. Both groups of treatment were compounded by statin-treated subjects, so this effect cannot be observed. But, as Cilla and colleagues found in their study, hypercholesterolemic subjects have higher EPHS levels than normocholesterolemic ones. In addition, basal endothelium cell adhesion levels for hypercholesterolemic patients were 11.7 ± 1.8 cells/mm^2^ (in line with our present results and significantly higher than that obtained in healthy donors, 5.5 ± 0.8 cells/mm^2^) [6]. The hypothesis of the present work was that if statins are unable to reduce the EPHS despite their cholesterol-lowering activity, PS may reduce both. Again, as no significant reduction in TC was observed (only a trend), it is plausible that no effect was observed regarding any of the eryptosis parameters determined. Nevertheless, our previous ex vivo study evaluating the PS effect on eryptosis-related parameters showed positive effects [10], but that study was conducted with blood from eight healthy volunteers. That ex vivo evaluation could not perfectly mimic the biological situation, since it did not consider the digestive process, interactions with the food matrix (bioaccessibility), and interindividual absorptive capacity (bioavailability). RBCs were stressed after blood extraction and then treated with PS, whereas in the present study, the oxidative stress was physiological. In addition, the PS concentration used by the authors was compatible with the chronic consumption of a PS-enriched beverage, not a food supplement, which could vary the serum concentration and, therefore, the biological activity.

No changes were observed after stratifying by sex, age, or BMI in terms of the eryptotic parameters. On the other hand, regarding potential correlations between variables, the no-change situation in EPHS remained whether the subjects were classified regarding physical activity or adherence to the Mediterranean Diet. In addition, no correlation was found between diet or physical activity and the outcome of adhesion to the endothelium.

It is worth noting that the small sample size (13 participants per group) could be responsible for the difficulty of detecting statistically significant changes. A higher sample would be necessary to confirm that the PS food supplement possesses a synergic hypocholesterolemic effect with low-to-moderate intensity statins and an effect on eryptosis. Nevertheless, in the present study, the broad and rigorous exclusion criteria, as well as the inclusion criteria, made it extremely difficult to enroll volunteers for the RCT.

Taking this into account, additional studies with a larger and more diverse population will be necessary to explore this topic in greater depth. These studies could provide a better understanding of the effects of the joint use of PSs and statins on CV health parameters. In this sense, using enriched foods could be a good option, since is easier to include them in the regular diet and some of them have good nutritional profiles.

## 5. Conclusions

With these results, we can conclude that the 6-week treatment with a PS supplement does not improve cholesterol blood levels or eryptosis markers in statin-treated hypercholesterolemic subjects. However, this intervention may improve cardiovascular health parameters such as an increase in Apo A1 and a subsequent reduction in the endothelial cell adhesion of RBC. These parameters are key for reducing atherosclerosis and therefore, reducing the risk of major cardiovascular events such as acute myocardial infarction or stroke. For this reason, but taking into account the limitations of the study, PS supplementation could be a beneficial dietary strategy for hypercholesterolemic subjects both under pharmacologic treatment and without treatment. Nevertheless, further studies with a wider population will be needed to delve deeper into the subject. In addition, studies using a food enriched with PSs could be of interest, as they are widely used, and it is easy to follow their consumption within the context of a normal diet.

## Figures and Tables

**Table 1:** Biochemical parameters before (T0) and after (T1) the treatment with placebo (control) or plant sterol-supplement (case).

	Control T0 (n = 13)	Control T1 (n = 13)	Case T0 (n = 13)	Case T1 (n = 13)
Glucose (mg/dL)	97.9 ± 10.0	93.7 ± 12.6 *	95.5 ± 9.8	93.9 ± 8.4
Insulin (µU/mL)	9.0 ± 4.5	8.0 ± 3.1	10.0 ± 5.3	9.8 ± 5.4
HOMA index	2.3 ± 1.3	1.9 ± 1.1	2.4 ± 1.4	2.3 ± 1.4
TC (mg/dL)	173.1 ± 25.9	165.2 ± 27.9	172.2 ± 28.2	168.1 ± 34.8
HDL-c (mg/dL)	53.8 ± 13.1	55.0 ± 13.2	55.5 ± 8.8	56.2 ± 10.4
LDL-c (mg/dL)	101.3 ± 22.7	92.0 ± 24.6 *	98.6 ± 25.7	94.9 ± 28.7
TG (mg/dL)	89.9 ± 24.9	91.6 ± 31.9	90.8 ± 32.9	84.6 ± 25.9
Apo A1 (mg/dL)	172.1 ± 30.1	177.1 ± 31.8	163.7 ± 19.4	174.1 ± 21.2 *
Apo B (mg/dL)	86.9 ± 15.2	84.2 ± 17.2	78.6 ± 18.6	83.6 ± 18.8
hsCRP	2.7 ± 2.2	2.1 ± 1.1	1.5 ± 1.0	1.8 ± 0.8

* indicates statistically significant differences (*p* < 0.05) compared to the basal levels from the same group. Apo: apolipoprotein; hsCRP: high sensitive C reactive protein; HDL-c: HDL cholesterol; HOMA: homeostasis model assessment; LDL-c: LDL cholesterol; TC: total cholesterol; TG: triglycerides. Data are expressed as mean ± standard deviation.

**Table 2:** Levels of adherence to the Mediterranean Diet following the MEDAS questionnaire criteria, and levels of physical activity following the IPAQ-Short questionnaire. Data are expressed as the number of subjects and percentage (in brackets).

**Adherence to the Mediterranean Diet**	**Low**	**Moderate**	**High**
Total (n = 26)	0	19 (73.1)	7 (26.9)
**Level of physical activity**	**Low**	**Moderate**	**High**
Total (n = 26)	14 (53.8)	9 (34.6)	3 (11.5)

## Data Availability

The original contributions presented in this study are included in the article. Further inquiries can be directed to the corresponding author.
